# Generation of pancreatic progenitors from human pluripotent stem cells by small molecules

**DOI:** 10.1016/j.stemcr.2021.07.021

**Published:** 2021-08-26

**Authors:** Yuqian Jiang, Chuanxin Chen, Lauren N. Randolph, Songtao Ye, Xin Zhang, Xiaoping Bao, Xiaojun Lance Lian

**Affiliations:** 1 Department of Biomedical Engineering, Pennsylvania State University, University Park, PA 16802, USA; 2 Huck Institutes of the Life Sciences, Pennsylvania State University, University Park, PA 16802, USA; 3 Department of Biology, Pennsylvania State University, University Park, PA 16802, USA; 4 Davidson School of Chemical Engineering, Purdue University, West Lafayette, IN 47907, USA; 5 Department of Chemistry, Pennsylvania State University, University Park, PA 16802, USA

**Keywords:** human pluripotent stem cells, Wnt signaling, definitive endoderm, pancreatic progenitors, pancreatic β cells, BMP signaling, small molecule differentiation, type 1 diabetes, induced pluripotent stem cells

## Abstract

Human pluripotent stem cell (hPSC)-derived pancreatic progenitors (PPs) provide promising cell therapies for type 1 diabetes. Current PP differentiation requires a high amount of Activin A during the definitive endoderm (DE) stage, making it economically difficult for commercial ventures. Here we identify a dose-dependent role for Wnt signaling in controlling DE differentiation without Activin A. While high-level Wnt activation induces mesodermal formation, low-level Wnt activation by a small-molecule inhibitor of glycogen synthase kinase 3 is sufficient for DE differentiation, yielding SOX17^+^FOXA2^+^ DE cells. BMP inhibition further enhances this DE differentiation, generating over 87% DE cells. These DE cells could be further differentiated into PPs and functional β cells. RNA-sequencing analysis of PP differentiation from hPSCs revealed expected transcriptome dynamics and new gene regulators during our small-molecule PP differentiation protocol. Overall, we established a robust growth-factor-free protocol for generating DE and PP cells, facilitating scalable production of pancreatic cells for regenerative applications.

## Highlights

• Differential activation of the Wnt pathway in hPSCs leads to mesoderm or endoderm fate
• BMP signaling inhibition enhances CHIR99021-induced DE differentiation from hPSCs
• Small-molecule DE cells can further become pancreatic progenitors and β cells
• RNA-seq of small-molecule differentiation revealed expected transcriptome changes

## Graphical abstract

**Figure:**
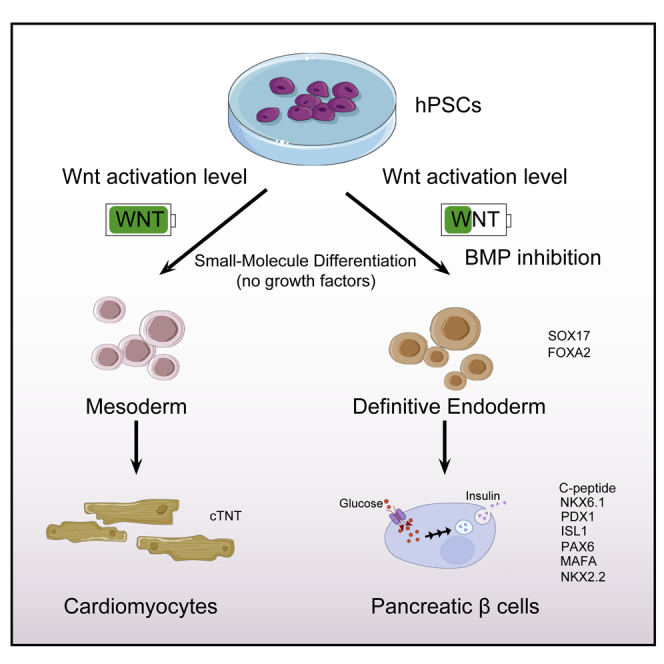

## Teaser

In this article, Lian and colleagues identify a dose-dependent role for Wnt signaling in differentiating hPSCs. While high-level Wnt activation induces mesoderm, low-level Wnt activation is sufficient to yield definitive endoderm (DE) cells. BMP signaling inhibition further enhances this differentiation. The authors further establish a small-molecule protocol to differentiate DE cells into pancreatic progenitors, which could be further differentiated into functional β cells.

## Introduction

Human pluripotent stem cells (hPSCs), including human embryonic stem cells (hESCs) (Thomson et al., 1998) and human induced pluripotent stem cells (iPSCs) (Takahashi and Yamanaka, 2006; Yu et al., 2007), can proliferate virtually indefinitely while maintaining the capacity to differentiate to a broad diversity of cell types. Because of these two unique properties, hPSCs are widely used as an *in vitro* model to study human development (Murry and Keller, 2008) and appreciatory cell sources for cell-based therapies (Randolph et al., 2017).

During human embryonic development, epiblast cells undergo the epithelial-mesenchymal transition to generate mesendoderm cells (Shook and Keller, 2003), which then give rise to the mesoderm or endoderm. This temporal and spatial determination of cell fate from epiblast cells depends on signaling cues in the surrounding environment, such as ligands from fibroblast growth factor (FGF) (Yu et al., 2011); the transforming growth factor (TGF)-β superfamily, including Activin/Nodal signaling and bone morphogenetic protein (BMP) signaling (Laflamme et al., 2007); and Wnt signaling (Lian et al., 2012). For example, in *Xenopus*, presumptive ectoderm can be re-specified into either mesoderm or endoderm by *Nodal* expression (Schier, 2003; Whitman, 2001). Similarly, during murine development, *Nodal* mutant embryos fail to form the mesendoderm and primitive streak (Conlon et al., 1994; Zhou et al., 1993). Using a hPSC differentiation model, researchers discovered that definitive endoderm (DE) differentiation of hPSCs is induced by high-dose Activin A treatment for multiple days (D'Amour et al., 2005; Xu et al., 2011).

In addition to the central role of Activin/Nodal signaling in endoderm differentiation, there is accumulating evidence that Wnt signaling plays a critical role in endoderm development. For instance, *Wnt3a* mutant mouse embryos lack the ability to form the primitive streak structure and also lack *Nodal* expression (Liu et al., 1999). Therefore, it is speculated that Wnt activation could induce *Nodal* expression. Indeed, NODAL expression in differentiated cells was reported upon activation of Wnt signaling in hPSCs (Lian et al., 2012). Because Wnt signaling is important in both mesoderm and endoderm development, it is unclear how pluripotent cells interpret Wnt signaling in two opposing ways: promoting mesoderm or endoderm differentiation. Here we systematically investigate Wnt signaling activity during hPSC differentiation and discover a dose-dependent Wnt signaling mechanism that controls mesoderm or endoderm specification. While high-level activation of the Wnt pathway induces mesoderm formation (Lian et al., 2012), low-level Wnt activation is sufficient to drive multiple hPSC lines to differentiate into DE cells, in part by directly inducing NODAL expression. Furthermore, we demonstrate that Wnt activation coupled with inhibition of BMP signaling further enhances DE differentiation, yielding over 85% SOX17^+^FOXA2^+^ DE cells in 4 days. These DE cells could be further differentiated into pancreatic progenitors (PPs) and β cells in the absence of growth factors. Our approach eliminates the growth factor requirement to produce DE cells and PPs, which could further be differentiated into functional β cells.

## Results

### Differential activation of Wnt pathway leads to distinct cell fates in hPSCs

**Figure 1:**
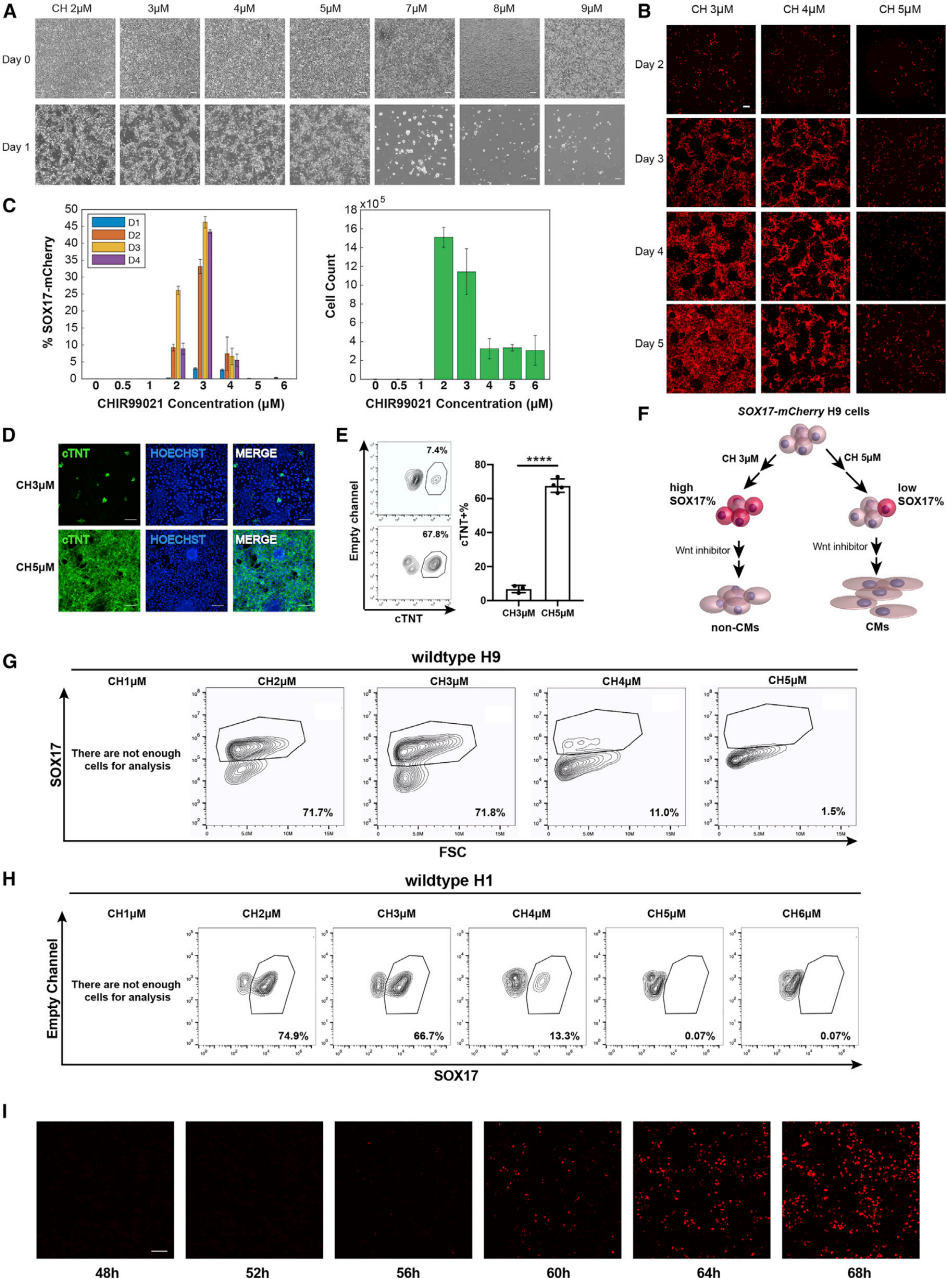
Activation of Wnt pathway to different levels leads to distinct cell fates in hPSCs On day 0, *SOX17-mCherry* knockin H9 cells were treated with CH at different concentrations in RPMI medium for 24 h and then cultured in RPMI plus B-27 minus insulin supplement for another 48 h without CH. On day 3, cells were treated with 2 μM Wnt inhibitor C59 for 48 h and then the medium was changed to the RPMI plus B-27 supplement. (A) Representative bright-field images were taken on day 0 and day 1. Scale bar, 100 μm. (B) Representative mCherry images of cells treated with CH at 3, 4, or 5 μM were taken from day 2 to day 5. Scale bar, 100 μm. (C) Flow cytometry analysis of mCherry expression and number of cells in *SOX17-mCherry* H9 cells from day 1 to day 4. Error bars represent SEM of three independent replicates. (D and E) *SOX17-mCherry* knockin H9 cells treated with CH at 3 or 5 μM on day 0 were analyzed on day 13 for cTNT expression by immunostaining (D) or flow cytometry (E). Scale bar, 100 μm. Error bars represent the SD of three or four independent experiments. ^∗∗∗∗^p < 0.0001, 3 μM CH versus 5 μM CH; Student's t test. (F) Diagram summarizing that *SOX17-mCherry* knockin H9 cells treated with 3 μM CH had high percentage of SOX17-expressing cells and became non-cardiomyocytes (CMs), while 5 μM CH induced low SOX17 expression and CM cell fate. (G and H) Unmodified H9 cells (G) or H1 cells (H) were treated with CH at different concentrations similar to the cells in (D). On day 4, the cells were collected for flow cytometry against SOX17. (I) RUES2_GLR cells (*SOX17-tdTomato*) were treated with 3 μM CH in RPMI for 24 h and then cultured in RPMI with B-27 minus insulin supplement. Time-lapse imaging was performed from day 2 to day 3 of differentiation. Representative images were shown at indicated time points. Scale bar, 100 μm. See also Figures S1 and S2 and Videos S1 and S2.

To probe how the differentiation trajectories can be affected by differential activation of the canonical Wnt pathway, we treated a *SOX17-mCherry* knockin H9 line (Ng et al., 2016) with a glycogen synthase kinase 3 (GSK3) inhibitor, CHIR99021 (CH), at different concentrations in RPMI medium for 24 h (Figure 1A). We noticed that CH at the highest concentration was toxic to hPSCs, thus causing massive cell death. Cells treated with CH at a very low concentration (<2 μM) also underwent apoptosis because of insufficient differentiation of hPSCs in RPMI. For example, for the H9 cells, only cells treated with 2–5 μM CH survived after 24-h CH treatment (Figure 1A). To investigate whether these CH-treated cells show distinct differential potential, we continued culturing the cells as in our GiWi protocol (Lian et al., 2012), that is, in RPMI with B-27 minus insulin supplement for 2 days, then with treatment with a Wnt inhibitor (Wnt-C59) for 2 days, followed by culture in RPMI with B-27 supplement for another 8 days (Figures 1B–1F and S1). Interestingly, during differentiation, whereas many mCherry^+^ cells were observed in the 3 μM CH treatment condition, few mCherry^+^ cells were generated in the 5 μM CH treatment condition (Figures 1B and S1). Furthermore, 3 μM CH treatment-induced cells retained mCherry expression on day 9 of differentiation, whereas most of the cells in the 4 or 5 μM CH treatment lost mCherry expression after day 7 (Figure S1), indicating that cells treated with different CH concentrations may vary in their differentiation trajectories. To quantify the percentage of SOX17^+^ cells upon CH treatment, we performed flow cytometry analysis of mCherry expression with cells from day 1 to day 4. It turned out that the maximum percentage of mCherry^+^ cells was achieved with 3 μM CH treatment (Figure 1C). The maximum number of cells was achieved with 2–3 μM CH treatment (Figures 1C and S1B). Eventually, while 5 μM CH treatment-derived cells robustly yielded cardiomyocytes, 3 μM CH treatment-derived cells produced few cTNT^+^ cardiomyocytes as shown by cTNT immunostaining (Figure 1D). Flow cytometry analysis of day 13 differentiated cells revealed that 67.6% ± 3.9% of cTNT^+^ cells were generated under 5 μM CH treatment. However, 3 μM CH treatment produced only 6.8% ± 2.1% cTNT^+^ cardiomyocytes (p < 0.0001, t test, 3 versus 5 μM) (Figure 1E). In addition, spontaneous contracting cardiomyocytes were observed in the 5 μM CH treatment condition (Video S1). These results indicate that CH treatment at a high dose leads to mesodermal cells with low SOX17 expression and further robust cardiomyocyte differentiation, whereas CH at a lower dose can induce hPSCs to become endodermal populations with high SOX17 expression (Figure 1F). To understand whether other hPSC lines show a similar dose-dependent differentiation pattern, we treated wild-type H9 and H1 cells with CH at different concentrations. On day 4 of differentiation, cells were collected and analyzed for SOX17 expression by flow cytometry. As expected, for H9 and H1 cells, the maximum percentage of SOX17^+^ cells was obtained with 2 or 3 μM CH treatment, consistent with the trend observed in *SOX17-mCherry* H9 cells (Figures 1G and 1H). To study the kinetic patterns of SOX17 expression during CH-induced endoderm differentiation, we treated RUES2_GLR reporter cells, which contain a *SOX17-tdTomato* knockin cassette, with 3 μM CH in RPMI and then cultured the cells in RPMI plus B-27 minus insulin supplement. Time-lapse imaging was performed from day 2 to day 3 of differentiation (Figure 1I, Video S2). SOX17-tdTomato expression was observed at 60 h of differentiation. Together, these results demonstrate that hPSCs can be differentiated to distinct cell fates by different levels of Wnt signaling activation.

Video S1. Sox17-mCherry H9 cells were treated with 5 μM CH in RPMI for 24 h and then cultured in RPMI plus B-27 minus insulin supplement for 2 days On day 3, the cells were treated with 2 μM Wnt-C59 for 2 days, followed by medium change to RPMI plus B-27 supplement from day 5. Video of beating cardiomyocytes was taken on day 9.

Video S2. RUES2_GLR cells (SOX17-tdTomato) were treated with 3 μM CH in RPMI for 24 h and then cultured in RPMI with B-27 minus insulin supplement Time-lapse imaging was performed from day 2 to day 3 of differentiation.

### CH-induced DE differentiation is dependent on the low activity of insulin signaling

Recently, Li et al. reported a 3-day DE differentiation protocol using both Activin A and CH (Li et al., 2019). Specifically, they demonstrated that both Activin A and CH were required for DE differentiation since no SOX17^+^ cells were generated via CH treatment alone. To determine why CH alone induced SOX17 expression in our protocol, we compared our culture medium with the Li et al. culture medium (Li et al., 2019). While we used RPMI (without B-27 minus insulin supplement for 24 h and with for 3 days) as our basal medium, Li et al. used Advanced RPMI as their basal medium. Whereas RPMI has no insulin, Advanced RPMI contains 10 mg/L insulin. Multiple previous studies have pointed out the role of insulin in supporting self-renewal and pluripotency and in antagonizing mesendodermal differentiation (Anderson et al., 2017; Freund et al., 2008; McLean et al., 2007; Wang et al., 2007; Yu and Cui, 2016). In 2007, McLean and colleagues demonstrated that two conditions were required for DE generation: signaling by Activin/Nodal family members and exclusion of inhibitory signaling generated by PI3K through insulin/IGF (McLean et al., 2007). To further investigate whether this is the reason for DE abolishment with CH alone in Advanced RPMI media as reported by Li et al., we treated *OCT4-GFP* H1 cells with CH and added 10 mg/L insulin at different times in insulin-free RPMI medium (Figure S2A). Insulin treatment throughout the entire differentiation process greatly restricted hPSCs from exiting pluripotency (Figure S2B). Under this condition, around 22% of the cells retained OCT4 expression, significantly higher than no insulin treatment (22.6% ± 7.3% versus 0.8% ± 0.2%, p < 0.001) (Figure S2C), and the fewest FOXA2^+^ (8.71%–10.3%) and SOX17^+^ (9.45%–12.2%) cells were generated (Figure S2C). Cells with no insulin treatment yielded 49.7%–51.7% SOX17^+^ and 48.7%–55.3% FOXA2^+^ cells, and 0.6%–1% OCT4^+^ cells. Our result was consistent with those of the Li et al. paper, in which they demonstrated that JNK-JUN signaling, which can be activated by insulin, functioned as a barrier to pluripotency exit (Li et al., 2019). In addition, unlike cells treated with insulin for 4 days, cells treated with insulin at a late stage of differentiation (e.g., from day 3 to day 4) did not show a statistically significant difference from the no-insulin-treated cells in GFP, FOXA2, or SOX17 expression, indicating that hPSCs in the first 2 days of differentiation are more sensitive to insulin treatment than during the late stage of differentiation (Figures S2B and S2C). This provides additional evidence that early stages of hPSC differentiation are of vital importance for pluripotency exit and lineage specification.

### Inhibition of BMP signaling enhances CH-induced DE differentiation from hPSCs

**Figure 2:**
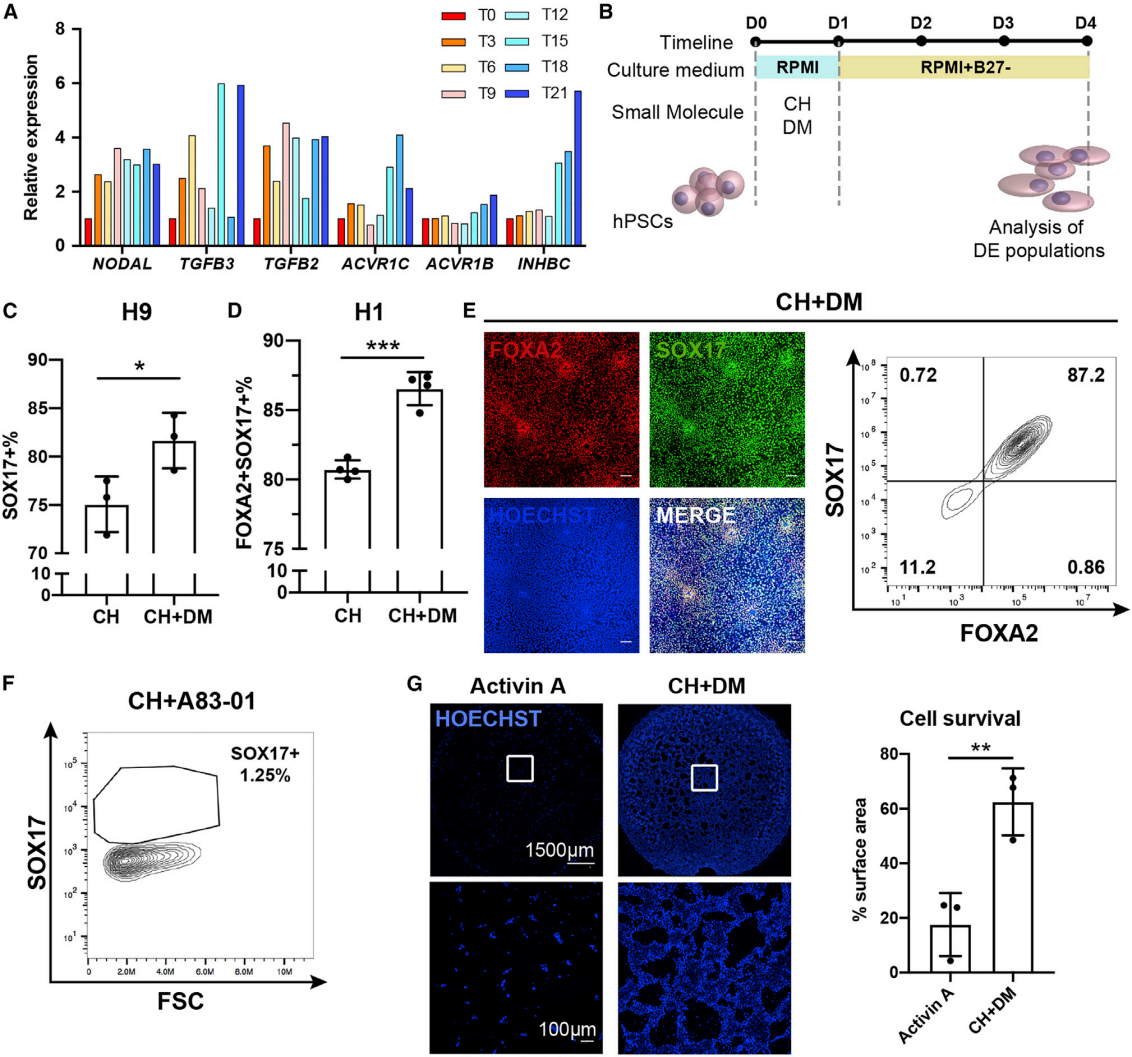
BMP inhibition enhances CH-induced DE differentiation (A) Human iPSC 19-9-11 cells treated with CH in RPMI were collected for RNA sequencing every 3 h for 21 h. Relative expression of selected TGF-β signaling-related genes is shown. (B) Schematic of protocol for differentiation of hPSCs to DE cells via treatment with small molecules (CH and DM). (C and D) Wild-type H9 cells (C) or *OCT4-GFP* H1 cells (D) were treated with CH alone or with CH plus DM as indicated in (B). On day 4 of differentiation, the cells were analyzed for FOXA2 and SOX17 expression by flow cytometry. Error bars represent SD of four independent experiments. ^∗^p < 0.05, ^∗∗∗^p < 0.001; Student's t test. (E) Representative images of immunostaining and plot of flow cytometry against FOXA2 and SOX17 on day 4 under CH plus DM condition. Scale bar, 100 μm. (F) *OCT4-GFP* H1 cells were differentiated with CH and A83-01 on day 0. On day 4, cells were analyzed for SOX17 expression by flow cytometry. (G) H9 cells were treated with 100 ng/mL Activin A in RPMI containing 0.2% FBS, or CH plus DM in RPMI for 24 h. On day 1, cells were stained with Hoechst. Scale bars are as indicated. Hoechst-positive areas were quantified in ImageJ. Error bars represent SD of three independent experiments. ^∗∗^p < 0.01; Student's t test. See also Figure S3 and Tables S1 and S2.

To understand the mechanism underlying CH-induced DE differentiation, we collected hPSCs treated with CH within 24 h for bulk RNA-sequencing (RNA-seq) analysis. Several TGF-β signaling ligands, including *NODAL*, *TGFB3*, and *TGFB2*, were significantly upregulated upon CH treatment, indicating that CH-treated cells produced endogenous Nodal/TGF-β signaling ligands, which may play critical roles in DE differentiation (Figures 2A, S3A, and S3B, Table S1). This finding is consistent with previous publications (Funa et al., 2015; Kozmikova and Kozmik, 2020; Lian et al., 2012). Activation of β-catenin via CH treatment promoted a sustained increase in NODAL levels and SMAD2 phosphorylation. In addition, CH treatment along with SB431542, which is an inhibitor of ALK5, ALK4, and ALK7, reduced SMAD2 phosphorylation (Funa et al., 2015; Kozmikova and Kozmik, 2020; Lian et al., 2012). These results provided evidence of the cross talk between canonical Wnt signaling and Nodal/TGF-β signaling.

We next hypothesized that enhancing endogenous Nodal signaling with other small molecules could further promote CH-induced DE differentiation. BMP signaling is a parallel pathway for Nodal/TGF-β signaling and has been reported to contribute to preimplantation development in both human and mouse (Coucouvanis and Martin, 1999; De Paepe et al., 2019). Since the BMP and Nodal pathways are competitive in SMAD4 recruitment, we assumed that Nodal signaling could be upregulated by blocking the BMP pathway, and this in turn would enhance DE differentiation. Therefore, we applied dorsomorphin (DM), an inhibitor of the BMP ALK2 receptor, together with CH to promote DE differentiation (Figure 2B). It turned out that DM addition led to an increase in the FOXA2^+^SOX17^+^ DE population with up to 87% efficiency in H9 and H1 cells based on dual expression of SOX17 and FOXA2, two markers of DE (Figures 2C–2E). Moreover, inhibition of BMP signaling also enhanced DE differentiation in the RUES2_GLR line (Figure S3C). Similarly, 6-9-9 iPSCs can be induced to generate 84.1% SOX17^+^ cells with CH plus DM treatment (Figures S3D and S3E). Considering that a GSK3 inhibitor and a BMP inhibitor are used for DE differentiation, we named our new DE differentiation protocol the GiBi protocol.

To test whether endogenous TGF-β signaling ligands are critical for CH-induced DE differentiation, we treated hESCs with CH plus a Nodal pathway inhibitor, A83-01. Flow cytometry against SOX17 on day 4 showed that the addition of A83-01 severely blocked CH-induced DE differentiation (Figure 2F), which supported the idea that CH-induced DE differentiation relies on endogenous Activin/Nodal signaling. We also tested various initial cell densities on DE differentiation efficiency, and found that 80% is the optimal confluency to start the differentiation (Figure S3F).

Activin A-based DE protocols were reported to have an issue with massive cell death during differentiation (Wang et al., 2015). We compared the cell viability of our GiBi protocol with that of Activin A-based conditions (100 ng/mL Activin A plus 0.2% FBS in RPMI) (D'Amour et al., 2005) and found a significant increase in cell survival with our GiBi protocol, while treatment with Activin A alone led to massive cell death in 24 h, as indicated in images of nuclear staining by Hoechst (Figure 2G). Therefore, our GiBi protocol increases the yield of differentiation by enhancing cell survival, in a serum-free and growth-factor-free system, compared with previous Activin A-based protocols. It is estimated that the cost of small molecules in the GiBi protocol is merely 1% that of Activin A in previous growth factor-based protocols (Table S2).

We also tried GiBi DE differentiation in a suspension system. H1 *OCT4-GFP* cells were seeded into mTeSR1 medium supplemented with 0.02% gellan gum for suspension culture. With the GiBi protocol, there were 45.3% SOX17^+^GFP^−^ cells generated on D4 (Figure S3G). There were 13% GFP^+^ undifferentiated cells on day 4, which we believe can be reduced by optimizing the drug concentration in the 3D system as well as the seeding cell density to control the cluster size. This result indicated that the GiBi protocol can also be applied for suspension differentiation.

### Small-molecule DE cells are capable of further differentiation toward pancreatic progenitors and β cells

**Figure 3:**
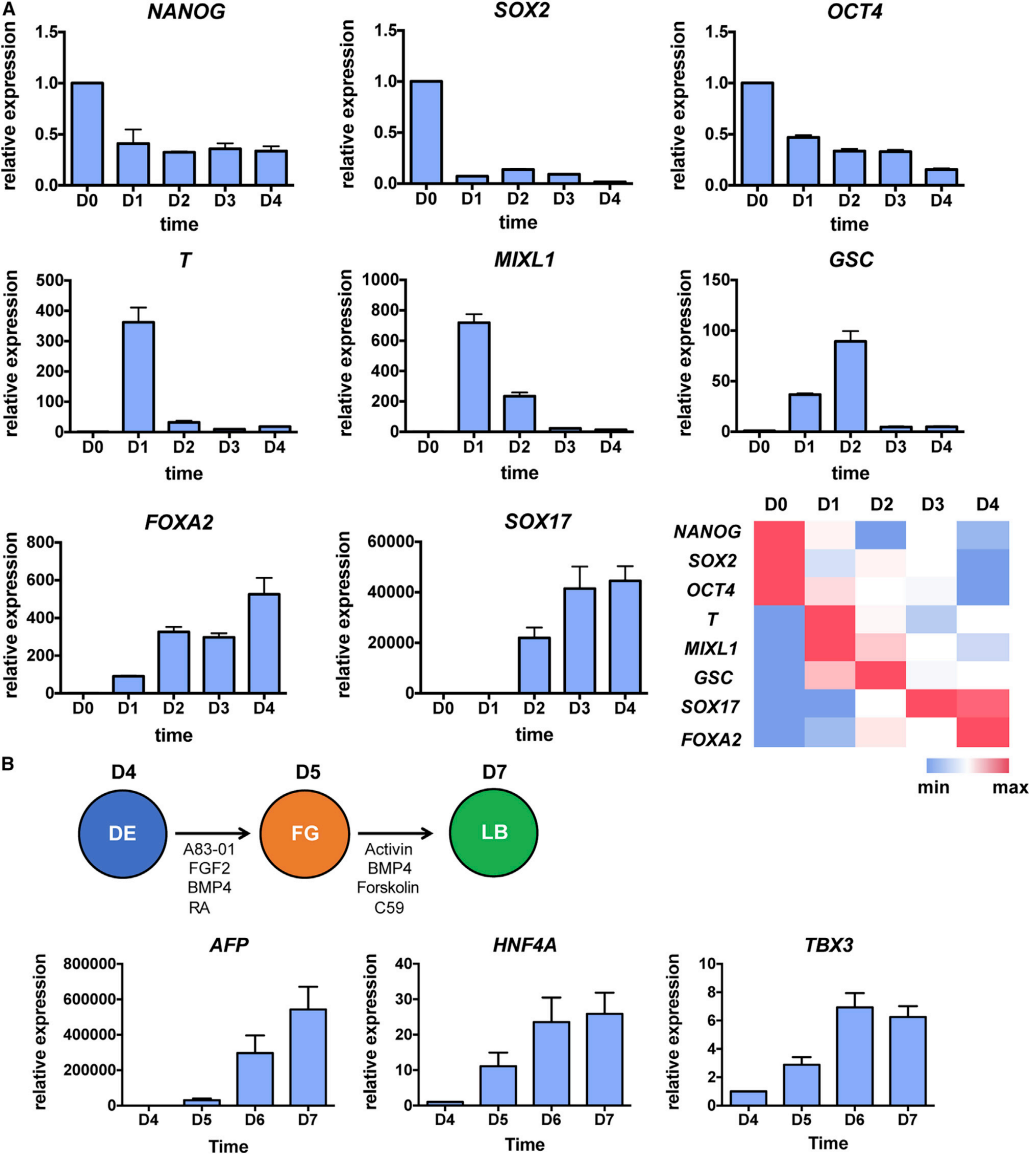
Characterization of DE cells generated from hPSCs via a GiBi protocol (A) *OCT4-GFP* H1 cells were differentiated with CH plus DM. At different time points, mRNA was collected, and qPCR analysis of pluripotent, mesendoderm, and endoderm gene expression was performed. Error bars represent SD of three technical replicates. (B) DE cells generated from (A) were further differentiated into posterior foregut (FG) and liver bud (LB). At different time points during differentiation, mRNA was collected and qPCR analysis of the expression of liver bud markers *AFP*, *HNF4A*, and *TBX3* was performed. Error bars represent SD of three technical replicates.

**Figure 4:**
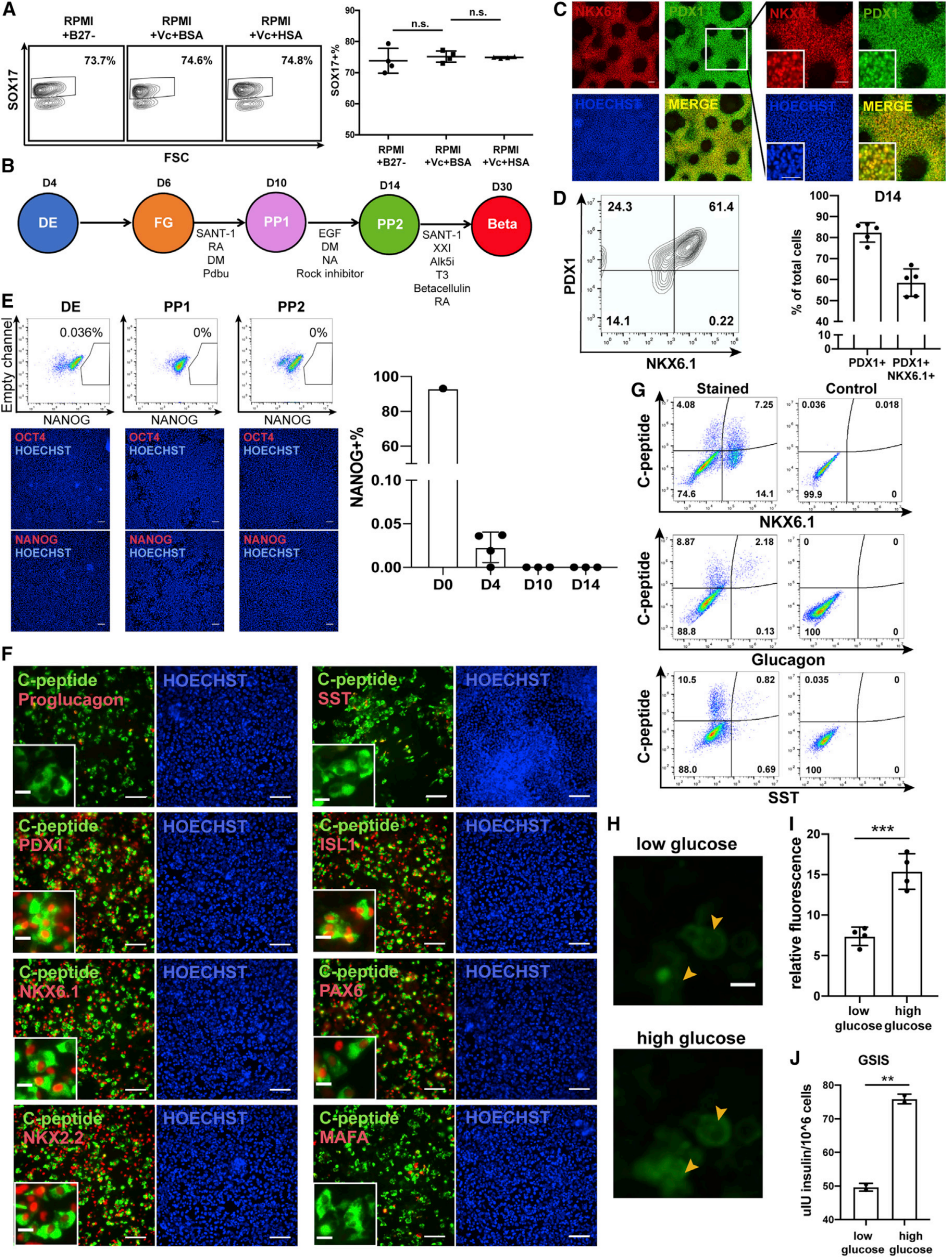
Robust generation of pancreatic progenitors and functional pancreatic β cells with small-molecule-derived DE cells (A) Flow cytometry against SOX17 with D4 cells treated with RPMI and B-27− or BSA + Vc or HSA + Vc. On the right is the quantification. n.s. indicates no significance. n = 4. (B) Diagram of the differentiation by which DE cells were further differentiated to posterior foregut (FG) and primary pancreatic progenitor cells (PP1), pancreatic progenitor cells (PP2), and pancreatic β cells (Beta). (C and D) Immunostaining images (C) and flow cytometry analysis (D) of D14 PP2 cells against NKX6.1 and PDX1. Scale bar, 100 μm; 50 μm for maximum magnification. (E) H1 wild-type cells were differentiated with the GiBi protocol to D4 and further differentiated to PP stages. Flow cytometry and immunostaining against NANOG and/or OCT4 was analyzed at the indicated time points. On the right is the quantification of NANOG flow data at the indicated time points. Scale bar, 100 μm. (F) Immunostaining with generated pancreatic β cells against C-peptide, proglucagon, SST, PDX1, ISL1, NKX6.1, PAX6, NKX2.2, and MAFA. Scale bar, 100 μm; 20 μm for maximum magnification. (G) Plots of flow cytometry with generated pancreatic β cells against C-peptide, NKX6.1, glucagon, and SST. (H) Fluo-4AM staining with generated pancreatic β cells under low- and high-glucose treatment. Scale bar, 20 μm. (I) Quantification of relative fluorescence intensity of the cells indicated by yellow arrows in (H). ^∗∗∗^p < 0.001. (J) GSIS analysis with generated pancreatic β cells from wild-type H1 line. ^∗∗^p < 0.01. See also Table S3.

We next characterized the GiBi-derived differentiated cell population by profiling the expression of key genes during DE differentiation and testing its capacity to differentiate into more specified lineages. Based on qPCR results, hPSCs lost the expression of genes related to pluripotency, like *NANOG* (Faddah et al., 2013), *SOX2* (An et al., 2019), and *OCT4* (Strebinger et al., 2019), over the course of differentiation (Figure 3A). Genes involved in gastrulation, like *T* (Nakanishi et al., 2009), *MIXL1* (Davis et al., 2008), or *GSC* (Soibam et al., 2015), were significantly upregulated only on day 1 or day 2 (Figure 3A). In contrast, DE-specific transcription factors, such as *FOXA2* (Lee et al., 2019) or *SOX17* (Xu et al., 2011), were gradually upregulated, demonstrating the successful generation of a DE population (Figure 3A). Using small-molecule DE cells, we further differentiated them toward liver or pancreatic lineages with published protocols (Ang et al., 2018; Nostro et al., 2015). qPCR analysis or immunofluorescence images revealed the upregulation of liver bud markers, such as *AFP*, *HNF4A*, and *TBX3* (Figure 3B), or PP genes like *PDX1* and *NKX6.1* (Figure 4), indicating the multipotency of the GiBi protocol-derived DE cells.

We next optimized the GiBi protocol medium to develop a xeno-free small-molecule-based DE protocol. We tested three media: RPMI basal medium supplemented with B-27 minus insulin, bovine serum albumin (BSA) plus ascorbic acid (Vc), or human serum albumin (HSA) plus Vc. We showed that RPMI with HSA plus Vc, which was xeno free, led to a similar yield of SOX17^+^ DE cells compared with the other two BSA-containing conditions (Figure 4A). Based on this result, we developed a modified protocol to derive DE cells and pancreatic cells (Figure 4B). Our xeno-free protocol generated up to 85% PDX1^+^ PP cells and 61% PDX1^+^NKX6.1^+^ PP2 cells on day 14 (Figures 4C and 4D) and worked broadly in several hESC and iPSC lines (Table S3). Compared with the publications over the past 5 years that rely on Activin A plus Wnt3a/CH for DE and pancreatic specification, our GiBi DE protocol generated up to 70% PDX1^+^NKX6.1^+^ PP2 cells by the end of stage 4, which was comparable to the PP2 efficiency of the Activin A-based protocols (Table S3). Together these results show that small-molecule DE induction as in our GiBi method will not have a negative impact on PP specification.

Analysis of *OCT4* and *NANOG* (Figure 4E) revealed rapid loss of pluripotency during the differentiation, consistent with the trend in qPCR results (Figure 3A). Furthermore, NKX6.1^+^ cells generated from our protocol were able to further specify into monohormonal pancreatic β cells, revealed by flow cytometry and immunostaining against β-cell-specific markers, including C-peptide, PDX1, NKX6.1, NKX2.2, ISL1, and PAX6 (Figures 4F and 4G). Minimal polyhormonal cells expressing glucagon or somatostatin (SST) were observed (Figures 4F and 4G). It is worth noting that differentiation of small-molecule PP2 cells into pancreatic β cells requires further head-to-head investigation and it is unclear that small-molecule PP2 cells generated here are the same as PP2 cells generated by other growth-factor-dependent protocols.

We then performed functional assays by staining the generated β cells with calcium dye Fluo-4AM and observed significant fluorescence increase under high-glucose treatment (Figures 4H and 4I). In addition, these hPSC-derived β cells secreted more insulin when treated with a higher concentration of glucose in a glucose-stimulated insulin secretion (GSIS) assay (Figure 4J), further supporting that our hPSC-derived β cells are functional.

**Figure 5:**
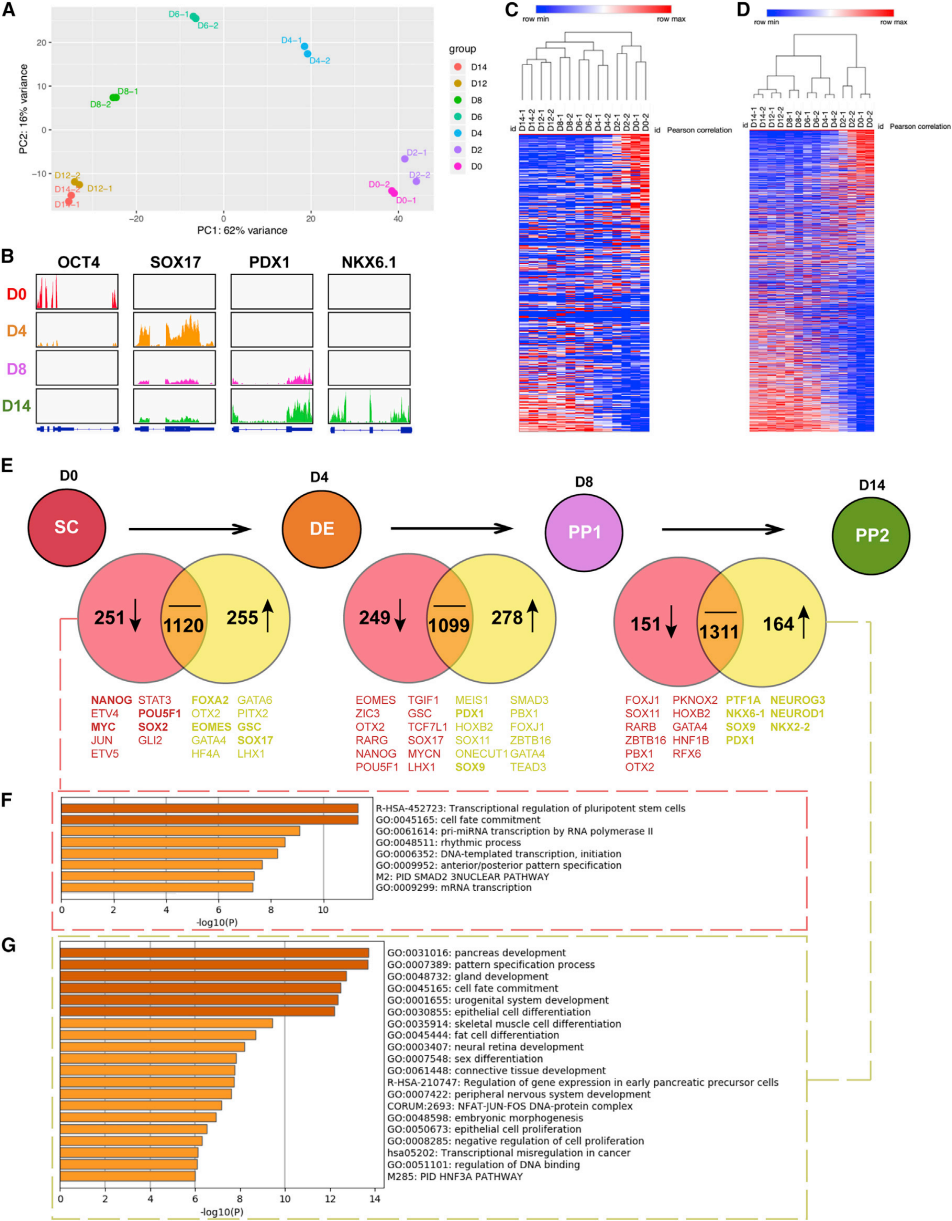
Characterization of hPSC-derived pancreatic differentiation with RNA sequencing (A) Stepwise developmental trajectory of hPSC-derived pancreatic progenitor cells reconstructed by principal-component (PC) analysis of RNA landscapes. The fraction of sample variance explained is indicated for each component. (B) Expression dynamics of key regulator genes, including *OCT4*, *SOX17*, *PDX1*, and *NKX6.1* on D0, D4, D8, and D14. Tracks display normalized RNA. Gene models are shown at the bottom. (C and D) Heatmaps of the whole transcriptome (C) and of the 1,639 transcription factors (D). (E) Venn diagrams of upregulated and downregulated genes of D4 compared with D0, D8 compared with D4, and D14 compared with D8. The false discovery rate is <0.05. The threshold of fold change is 2. (F and G) GO analysis of top 100 downregulated genes on D4 compared with D0 (F), and of top 100 upregulated genes on D14 compared with D8 (G). See also Figure S4.

To further investigate how hPSCs differentiate into PPs using our differentiation protocol, we performed RNA-seq to profile transcriptome dynamics with cells harvested at different time points, including day 0 (D0), D2, D4, D6, D8, D12, and D14. We confirmed good quality of RNA samples and sequencing data via quality control checks. Replicate transcriptome measurements showed high consistency and independently recapitulated the stepwise development of the hPSC-derived PP cells (Figure 5A). Mapping RNA-seq reads to key regulator transcription factors on each stage presented distinctive expression at different time points (Figure 5B). For instance, *OCT4*, which is crucial in pluripotency maintenance of hPSCs, showed high expression exclusively on D0, while *SOX17*, a critical DE marker gene, was transiently upregulated on D4. In addition, *PDX1* and *NKX6.1*, both PP marker genes, increased expression after D8 and D14, respectively, indicating successful induction of stepwise pancreatic specification. Heatmaps of the whole transcriptome (Figure 5C) and all transcription factors (Figure 5D) showed consistency in gene expression pattern, as well as in hierarchical clustering. Analysis of differential gene expression over different time points revealed a transition from hPSC, through DE, to PDX1^+^ PP and NKX6.1^+^ PP, marked by upregulation and downregulation of a series of stage-specific key regulator genes (Figures 5E and S4). Specifically, from D0 to D4, pluripotency-related genes comprising *NANOG*, *STAT3*, *MYC*, *POU5F1*, and *SOX2* decreased, while genes that are important in gastrulation and DE specification, like *GSC*, *EOMES*, *SOX17*, *GATA4*, *GATA6*, and *FOXA2*, were upregulated. This was followed by ascending expression of early pancreatic-related genes, like *SOX9*, *GATA4*, *ONECUT1*, and *PDX1*, along with further decreasing pluripotency or gastrulation-related genes such as *EOMES*, *GSC*, *SOX17*, *NANOG*, and *POU5F1* from D4 to D8. In the last transition from D8 to D14, representative genes of PP cells, like *NKX6.1*, *PTF1A*, *NKX2.2*, *NEUROD1*, and *NEUROG3*, were significantly upregulated. Gene ontology (GO) analysis indicated a trend of functional transitions across differentiation processes, which displayed downregulation of genes in pluripotency maintenance and upregulation in pancreatic specification (Figures 5F, 5G, and S4).

Together, our GiBi-derived DE cell population holds the potential to further specify into pancreatic lineages with expected transcriptome changes, which marks an alternative approach to current pancreatic differentiation protocols.

## Discussion

This study demonstrates efficient and robust generation of DE cells from multiple hPSC lines solely via small-molecule modulation of the Wnt and BMP signaling pathways. Furthermore, DE cells produced via small-molecule treatment could further differentiate into the liver bud lineage or PP and β cells. Using our GiWi protocol, we showed that activation of Wnt signaling via CH treatment at a high concentration promotes cardiomyocyte differentiation (Lian et al., 2012, 2015). Here we show that Wnt signaling activation via CH treatment at a low concentration, however, robustly and consistently generates SOX17^+^ and FOXA2^+^ DE cells. Our findings suggest that canonical Wnt signaling can act as a master regulator of both cardiac mesoderm and DE specification from pluripotent cells in a dose-dependent manner, with a low-level activation for efficient DE differentiation.

A recent publication from Li et al. (Li et al., 2019) showed that CH treatment alone was not sufficient to differentiate hPSCs into DE cells (0% SOX17^+^ cells generated), which is contrary to our observation. To understand this difference, we noticed that the Li et al. study used Advanced RPMI medium as their basal medium, which contains 10 mg/L insulin and 400 mg/L AlbuMAX II (one type of BSA). In our GiBi protocol, we use RPMI, which does not contain any insulin or BSA, as our basal medium during CH and/or DM treatment. We previously showed that BSA effectively inhibits CH-induced mesendoderm differentiation (Lian et al., 2015), which may partially explain why Li et al. did not achieve successful DE differentiation via CH-only treatment. In addition, we showed in this study that addition of insulin (10 mg/L) into RPMI medium greatly inhibited our CH-induced DE differentiation when included during the first 2 days of differentiation. Taken together, successful small-molecule DE differentiation is dependent on the absence of insulin signaling.

Although exogenous Activin/Nodal signaling growth factors are not necessary for CH-induced DE differentiation, a Nodal pathway inhibitor (A83-01) completely abrogated CH-induced DE differentiation, highlighting the importance of endogenous Activin/Nodal signaling in our differentiation. Expression of NODAL early in differentiation, when the majority of cells are either OCT4 or Brachyury positive, suggests that endogenous NODAL protein may be produced by undifferentiated cells or mesendoderm cells. Since endogenous activin/Nodal signaling is required for CH-induced DE differentiation and Activin/Nodal signaling competes with BMP signaling for the co-activator SMAD4, inhibition of BMP signaling was tested to enhance CH-induced DE differentiation. We demonstrated that BMP signaling inhibitors greatly facilitated CH-induced DE differentiation. The paradigm of modulating Wnt and BMP signaling pathways with just two small molecules, which enhance endogenous expression of DE-promoting growth factors, simplifies the DE differentiation from hPSCs.

Our small-molecule protocol enabled the generation of PDX1^+^NKX6.1^+^ PP cells, which could be further differentiated into β cells following protocols developed in the past decade. In 2014, generation of glucose-responsive monohormonal pancreatic β cells was independently reported by two groups (Pagliuca et al., 2014; Rezania et al., 2014). These hPSC-derived β cells not only expressed markers found in mature β cells, but also displayed GSIS. This was followed by many researchers exploring approaches to simplifying or modifying these β-cell-differentiation protocols, including YAP inhibition (Rosado-Olivieri et al., 2019), cell aggregation (Toyoda et al., 2015), and manipulation of the cytoskeleton (Ghazizadeh et al., 2017; Hogrebe et al., 2020; Toyoda et al., 2017) or gene profile (Matsuoka et al., 2017; Toyoda et al., 2015; Walczak et al., 2016), as well as identification of stage-specific surface markers (Cogger et al., 2017; Mahaddalkar et al., 2020; Veres et al., 2019). To date, functional β cells can be generated under both suspension (Pagliuca et al., 2014) and 2D (Hogrebe et al., 2020) culture conditions. Our small-molecule-based PP protocol, when coupled with published PP-to-β cell protocols, will eventually enable large-scale production of functional β cells in a robust and cost-effective way, facilitating translation of these cells to cell-based therapies for treating type 1 diabetes.

## Experimental procedures

### Maintenance and differentiation of hPSCs

hESCs (H9, H1, and *OCT4-GFP* H1) (Zwaka and Thomson, 2003) and human iPSCs (6-9-9, 19-9-11, IMR90C4) (Yu et al., 2009) were obtained from WiCell Research Institute. The HUES8 cell line was obtained from Harvard University. The RUES2_GLR reporter cell line was from Rockefeller University. H9 *SOX17-mCherry* reporter cells were obtained from Dr. Ed Stanley at Murdoch Children’s Research Institute. Human pluripotent stem cell work was approved by the Embryonic Stem Cell Oversight Committee at The Pennsylvania State University. Undifferentiated hPSCs were maintained on iMatrix-511 (Stemgent)-coated plates in mTeSR1 medium (STEMCELL Technologies). When cells were 80%–90% confluent (usually 3–4 days after passaging, daily monitoring is necessary), medium was aspirated and 1 mL Accutase (Innovative Cell Technologies) was added to each well. The cells were incubated at 37°C, 5% CO_2_, for 10 min. Dissociated cells were transferred into excess DMEM at a 1/2 (v/v) ratio and centrifuged at 1,000 rpm for 4 min. The cell pellet was resuspended in mTeSR1 with 5 μM Y-27632 and 0.75 μg/mL iMatrix-511, and 10,000–20,000 cells/cm^2^ were seeded into wells. Incubated at 37°C, 5% CO_2_, hPSCs were routinely tested for mycoplasma, and all the cells were negative for mycoplasma contamination. For DE and further differentiation, cells were cultured on iMatrix 511-coated wells throughout. Most of the data in this study were collected from cells cultured in 6-well plates (VWR #62406-161), but it also worked well in 12-well plates (VWR #62406-165) or 24-well plates (VWR #62406-183).

#### DE differentiation

DE differentiation was initiated when the hPSCs reached 70%–80% confluency. hPSCs were treated with RPMI (Gibco) containing CH (Cayman Chemical) at concentrations ranging from 2 to 8 μM and 1 μM DM (Sigma Aldrich) for 24 h. Different cell lines may require distinct optimal CH concentration for DE differentiation. Cells were then cultured for another 3 days in RPMI containing either 2% v/v B-27 minus insulin supplement (Thermo Fisher) or 0.05% HSA and 200 μg/mL Vc.

We have developed PP and liver bud differentiation protocols based on previously described protocols (Ang et al., 2018; Nostro et al., 2015).

#### Pancreatic progenitor differentiation

hPSCs were treated with CH of optimized concentration and 1 μM DM in RPMI for 24 h (day 0 to day 1) and then cultured in RPMI supplemented with 0.05% HSA and 200 μg/mL Vc for another 5 days (day 1 to day 6). From day 6 to day 10, cells were treated with 0.25 μM SANT-1, 2 μM retinoic acid (RA), 0.75 μM DM, and 0.2 μM phorbol 12,13-dibutyrate in DMEM containing 1% v/v B-27 and 50 μg/mL Vc. From day 10 to day 14, cells were treated with 0.75 μM DM, 10 mM nicotinamide, and 100 μM Y-27632 in DMEM containing 1% v/v B-27 and 50 μg/mL Vc. Epidermal growth factor at 100 ng/mL is optional for day 10 to day 14. For specification of PP cells into β cells, D14 PP2 cells were treated with stage 5 and 6 differentiation cocktails based on previously reported studies (Velazco-Cruz et al., 2019).

#### Liver bud differentiation

For liver bud differentiation (Ang et al., 2018), day 4 DE cells from the GiBi protocol were treated for 1 day with 1 μM A83-01, 10 ng/mL FGF10, 30 ng/mL BMP4, and 2 μM RA in CDM3 medium (IMDM/F12, 10% knockout serum replacement, 1% chemically defined lipid concentrate [Thermo Fisher, #11905031], and 1% penicillin-streptomycin). After that, cells were cultured with 10 ng/mL Activin A, 30 ng/mL BMP4, 1 μM forskolin, and 1 μM Wnt-C59 in CDM3 medium for 2 days.

#### Cardiomyocyte differentiation (GiWi protocol)

Differentiation started when cells were at least 80% confluent. On day 0, cells were treated with 3 or 5 μM CH in RPMI for 24 h, followed by medium change to RPMI plus B-27 without insulin supplement for 48 h. On day 3 of differentiation, the cells were treated with 2 μM Wnt-C59 in RPMI plus B-27 without insulin supplement for 2 days. From day 5, the cells were cultured in RPMI plus B-27 supplement, with medium change every 3 days.

### Immunostaining

Cells were fixed with 4% formaldehyde at room temperature for 15 min and then blocked in DPBS with 0.4% Triton X-100 and 5% non-fat dry milk for 1 h. After that, the cells were sequentially stained with primary and secondary antibodies. Nuclei were stained with Hoechst 33342 (Thermo Fisher). Images were captured using a Nikon Ti Eclipse epifluorescence microscope. Images were processed using ImageJ software.

### Quantitative polymerase chain reaction

Cells were lysed with TRI-Reagent for 1 min and RNA was extracted with a Direct-zol RNA MiniPrep Plus kit (Zymo Research). cDNA was synthesized with a Maxima First Strand cDNA Synthesis Kit (Life Technologies). Quantitative PCR was performed using the SYBR Green PCR master mix (Life Technologies) and run on a CFX Connect real-time qPCR machine (Bio-Rad). GAPDH was used as the housekeeping gene for reference. Data were analyzed with the ΔΔC_T_ method. Primers are listed in Table S4.

### Flow cytometry

Cardiomyocytes were dissociated with trypsin-EDTA (0.25%) (Thermo Fisher) for 5 min. Other differentiated cells were dissociated with TrypLE Express Enzyme for 9 min. For flow cytometry with live cells, cells were resuspended in FlowBuffer-1 (DPBS with 0.5% BSA). For flow cytometry analysis using fixed cells, 1% formaldehyde in DPBS was used to fix cells for 30 min. After that, the cells were stained with primary and secondary antibodies (Table S4) in FlowBuffer-2 (DPBS with 0.5% BSA and 0.1% Triton X-100). Most data were collected on a BD Accuri C6 Plus flow cytometer, and data for H9 *SOX17-mCherry* reporter cells were collected with a BD LSRFortessa flow cytometer. Data were processed in FlowJo software.

### Fluo-4AM assay

Cells were stained with 4 μM Fluo-4AM dye for 30 min in ESFM medium, followed by three washes. Then the cells were incubated in ESFM for 5 min and images were taken at 500ms exposure time. The cells were then incubated in ESFM supplemented with 20 mM glucose for another 5 min and images were taken at the same exposure time.

### Glucose-stimulated insulin secretion assay

GSIS assay was performed in KRB buffer containing 3 or 20 mM glucose as previously reported (Hogrebe et al., 2020). Insulin concentration was measured with a human insulin ELISA kit (Alpco, #80-INSHU-E01.1).

### RNA sequencing

For every-3-h analysis of CH-treated iPSCs, cells were lysed with TRI reagent for 1 min and RNA was extracted with a Direct-zol RNA MiniPrep Plus kit (Zymo Research), followed by RNA purification with an RNA Clean & Concentrator kit (Zymo Research). Samples were analyzed with an Agilent Bioanalyzer for quality control. mRNA libraries were generated with the TruSeq Stranded mRNA library kit. Data were collected with HiSeq 100-nt single-read sequencing. The RNA-seq reads were trimmed and mapped to the hg19 reference using HISAT2 (Kim et al., 2019). Expression levels for each gene were quantified using the Python script *rpkmforgenes* and annotated using RefSeq. Genes without at least one sample with at least 10 reads were removed from the analysis. Principle-component analysis was done and heatmaps were constructed using R and Gene-E, respectively.

For 14-day analysis of PP differentiation, cells from different time points of pancreatic differentiation were lysed with TRI reagent. Total RNA was extracted with the Direct-zol RNA MiniPrep kit (Zymo Research #2071) and further purified with an RNA Clean & Concentrator kit (Zymo Research #R1013). RNA quality was characterized with a Bioanalyzer. cDNA libraries were prepared with Illumina TruSeq Stranded mRNA library construction kit and sequenced using NextSeq High Output 75-nt single-read sequencing.

RNA-seq data were mostly analyzed in the Galaxy platform (usegalaxy.org). Fastq files were checked for quality control with FastQC and organized with MultiQC. Sequencing reads were mapped to the human hg38 reference genome assembly using Kallisto with default parameters. Differential gene expression was determined with DESeq2 and limma-voom. For dynamics of marker gene expression, reads were aligned to the human hg38 reference genome assembly and displayed in the IGV viewer. Heatmaps were generated with normalized count file in Morpheus (https://software.broadinstitute.org/morpheus/). Hierarchical clustering was performed using one minus Spearman rank correlation. GO analysis was performed in the Metascape platform with the top 100 upregulated or downregulated genes (http://metascape.org/gp/index.html#/main/step1). Fold change was filtered as larger than 2 or less than 0.5.

### Statistical analysis

Quantification of flow cytometry data is shown as the mean ± SD unless otherwise indicated. Unpaired two-tailed Student's t test was used for comparison between two groups. p ≥ 0.05 was considered not significant; ^∗^p < 0.05, ^∗∗^p < 0.01, ^∗∗∗^p < 0.001, ^∗∗∗∗^p < 0.0001 were considered significant.

### Data and code availability

Sequencing data are available under NCBI GEO accession no: GSE142572 and GSE146985.

## Author contributions

X.L.L. designed the experiments and analyzed the results. Y.J. and C.C. performed the experiments and analyzed data. L.N.R. performed flow cytometry analysis of *Sox17-mCherry* cells. S.Y., X.Z., and Y.J. designed and performed the time-lapse imaging experiment. Y.J. and X.L.L. wrote the manuscript. Y.J., X.B., and X.L.L. contributed to revision of the manuscript. X.L.L. supervised the experiments.

## Conflicts of interest

A patent has been filed on the basis of this work, on which X.L.L. and C.C. are named as inventors.

## Supplemental information

Document S1. Figures S1–S4 and Tables S1–S4

Document S2. Article plus supplemental information
