# Supplementary material for: Generation of pancreatic progenitors from human pluripotent stem cells by small molecules
Source: Stem Cell Reports. 2021 Aug 26;16(9):2395–409. doi: 10.1016/j.stemcr.2021.07.021 (PMC8452541; doi:10.1016/j.stemcr.2021.07.021)
Supplement: Document S1. Figures S1–S4 and Tables S1–S4 [file mmc1.pdf]

Stem Cell Reports, Volume *16*

Supplemental Information

Generation of pancreatic progenitors from human pluripotent stem cells by small molecules

Yuqian Jiang, Chuanxin Chen, Lauren N. Randolph, Songtao Ye, Xin Zhang, Xiaoping Bao, and Xiaojun Lance Lian

Fig.S1 (Related to Fig. 1)

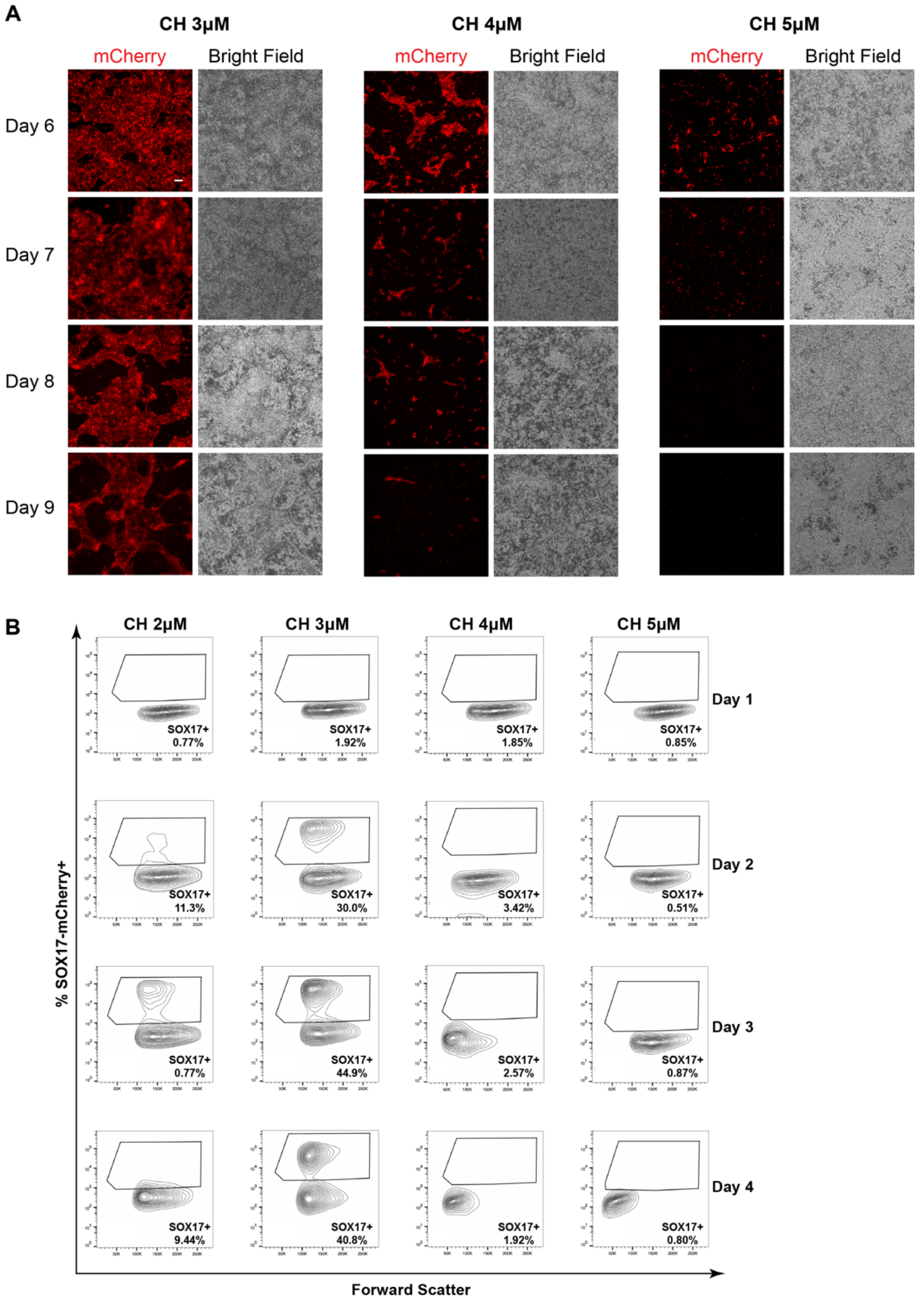

Fig. S1. SOX17-mCherry knockin H9 cells were treated with CH of concentrations from 3 μM to 5 μM in RPMI for 24 hours and then cultured in RPMI plus B-27 minus insulin supplement for two days. On day 3, cells were treated with 2 μM Wnt-C59 for two days, followed by medium change to RPMI plus B-27 from day 5. A. Representative mCherry and bright field images were taken from day 6 to day 9. Scale bar, 100 μm. B. Representative flow plots as in **Fig. 1C**.

Fig. S2 (Related to Fig. 1)

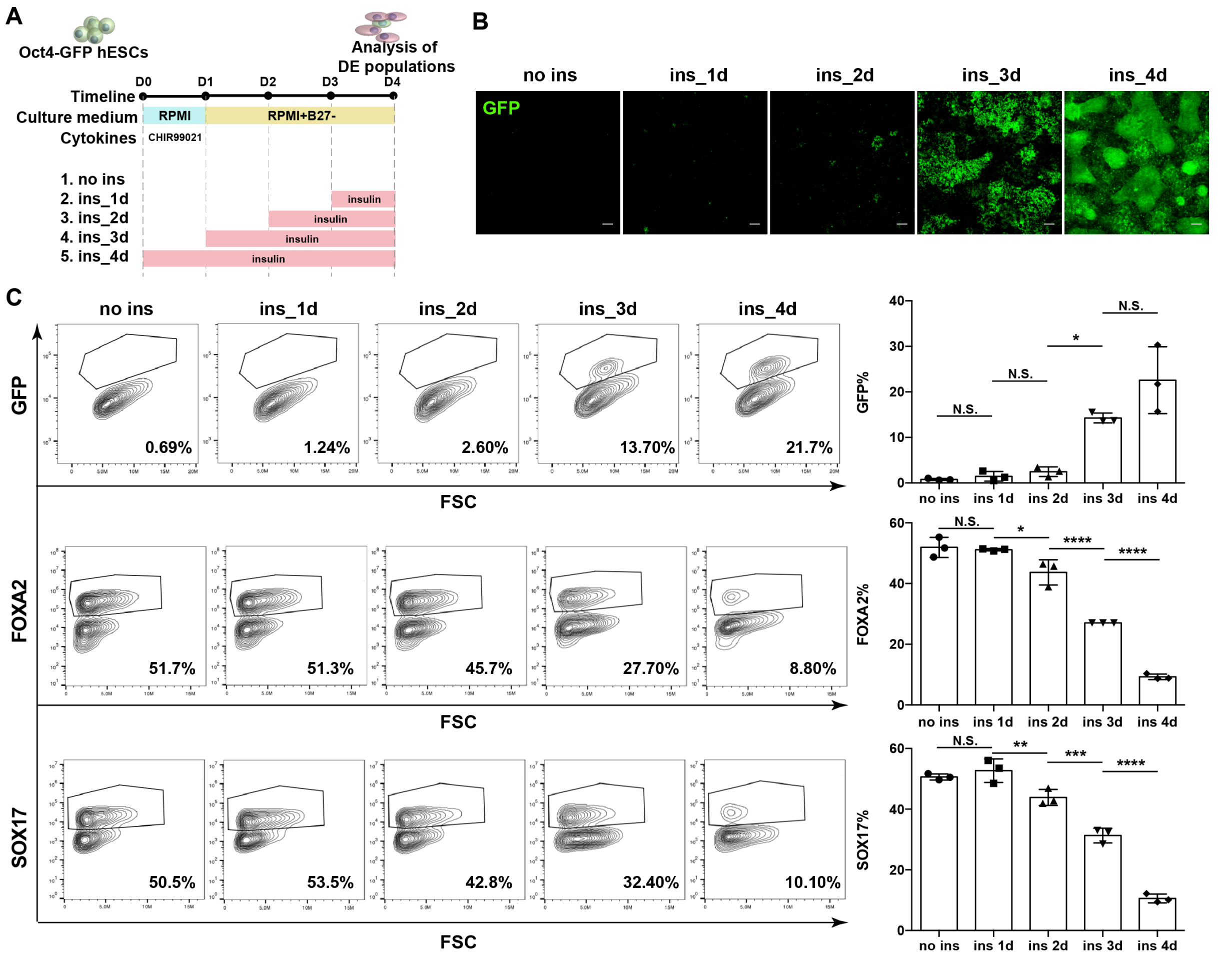

Fig. S2. Insulin inhibits CH-induced DE differentiation. (A-C) OCT4-GFP knockin H1 cells were treated with CH in RPMI for 24 hours and then cultured in RPMI plus B-27 minus insulin supplement for another 3 days. 10 μg/mL insulin was added with different durations as indicated (A). On day 4 of differentiation, GFP images were taken, showing the presence of OCT4 with insulin treatment. Scale bar, 100 μm (B). On day 4, cells were analyzed for GFP, FOXA2 and SOX17 expression by flow cytometry. Error bars represent SD of three independent experiments (C).

Fig. S3 (Related to Fig. 2)

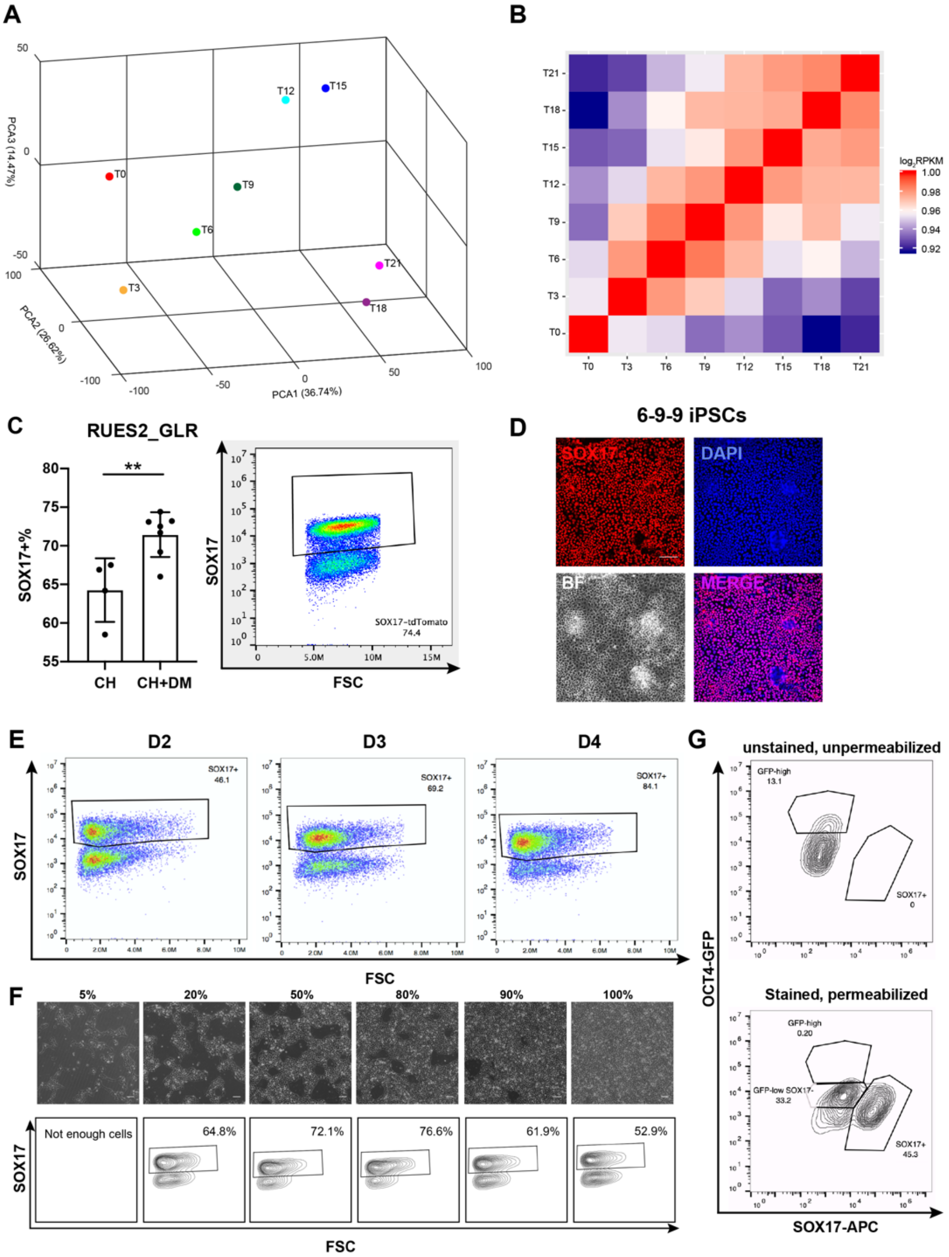

Fig. S3. (A) Human iPSC 19-9-11 cells were treated with 6 μM CH in RPMI medium. At different time points, cells were collected for RNA sequencing for every three hours until 21 hours. PCA projection of differentiated cell samples during differentiation, colored by indicated time points. (B) Correlation heatmap of whole transcriptome of eight different samples. (C) RUES2 SOX17-tdTomato cells were treated with 3 μM CH and 1 μM DM in RPMI for 24 hours and then cultured in RPMI plus B-27 minus insulin supplement for another 3 days. On day 4, cells were analyzed for SOX17-tdTomato expression via flow cytometry. (D,E) Human iPSC 6-9-9 cells were treated with 1 μM DM and 3 μM CH in RPMI for 24 hours and then cultured in RPMI plus B-27 minus insulin supplement for another three days. Cells were analyzed for SOX17 expression by immunostaining on day 4 (D) or by flow cytometry from day 2 to day 5 (E). (F) Human iPSC 6-9-9 cells were differentiated via the GiBi protocol. On day 0, cells were differentiated with different starting densities (from 5 to 100% confluency). On day 4 of differentiation, cells initiated at different densities were analyzed for SOX17 expression by flow cytometry. (G) OCT4-GFP H1 cells were differentiated with GiBi protocol in suspension system. On D4, cells were collected for flow cytometry stained against SOX17. There were 45.3% SOX17+GFP- DE cells and 13.1% GFP+ undifferentiated cells on D4.

Fig. S4 (Related to Fig. 5)

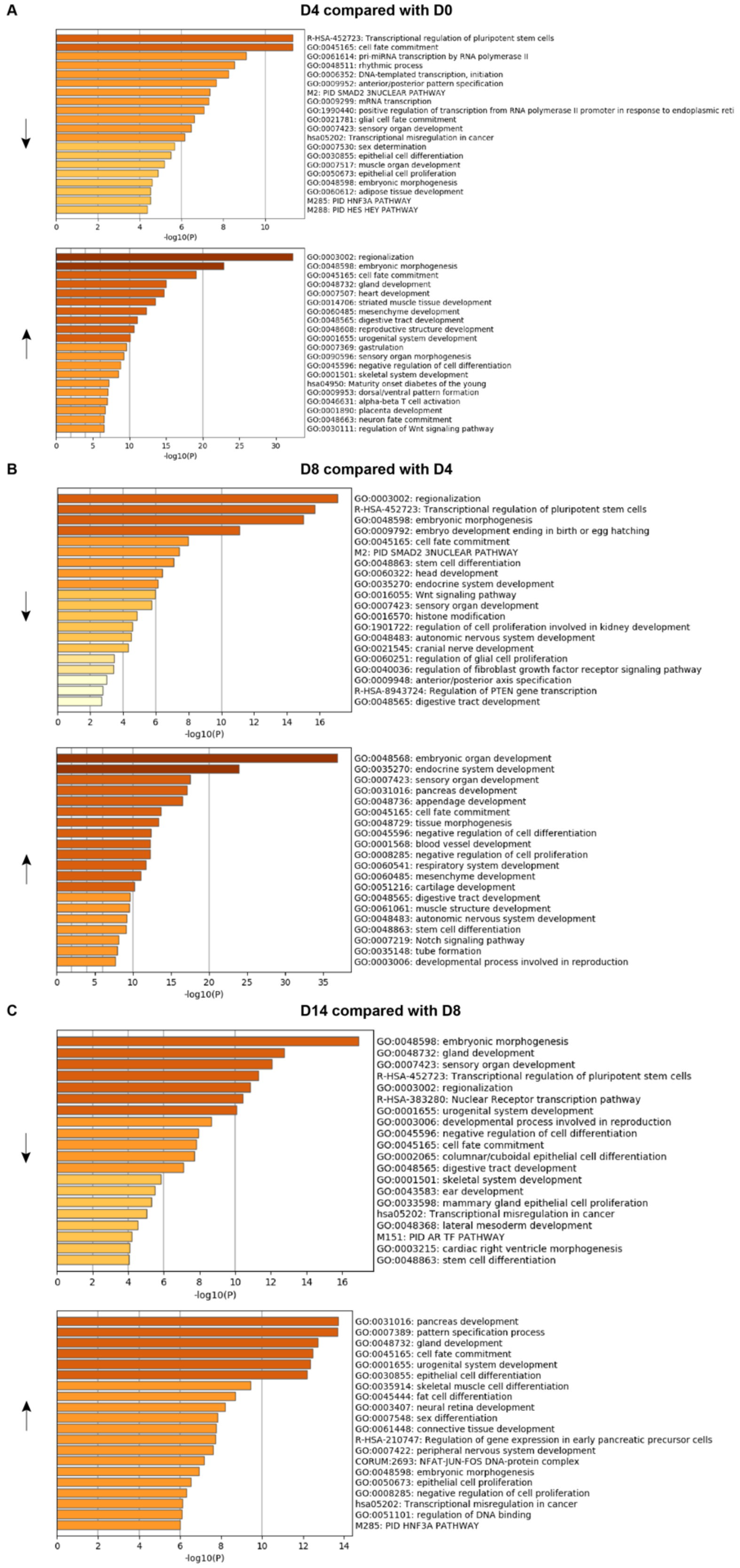

Fig. S4. Heatmaps listing the top statistically enriched terms with the top 100 genes that are most significantly downregulated or upregulated comparing D4 to D0 (A), D8 to D4 (B), and D14 to D8 (C). Filters of “FC greater than 2” or “FC less than 0.5” were applied to upregulated genes or downregulated genes, respectively.

Table S1: Expression of TGF-β ligands in CH-treated hPSCs for 0 h or 21 hours from bulk RNA-seq. Upregulated genes are highlighted in green and downregulated ones in orange.

	T0	T21
NODAL	40.5646035	121.910605
TGFB1	17.0573244	6.59393254
TGFB2	0.04640755	0.18715772
TGFB3	0.05501382	0.32540361
INHBA	1.11956025	0
INHBB	0.08315107	0.06387453
INHBC	0.09220856	0.52681492
INHBE	8.41511378	10.2001231
BMP2	2.59689663	1.0067133
BMP3	0.01327086	0
BMP4	3.10034327	9.44989031
BMP5	0.07013374	0.51854639
BMP6	1.04319278	0.75961687
BMP7	5.47446838	7.47355386
BMP8a	0.21697467	0.37636156
BMP8b	0.30907847	0.48474513
GDF3	19.7698081	38.6531585
GDF5	0	0.04390346
GDF6	0.08224276	0.08291953
GDF7	0.05724306	0.12825359
GDF9	0.37002775	0.81929691
GDF10	0.08624304	0
GDF11	5.01812216	6.12663683
GDF15	6.24614032	2.42950771
GDNF	0.08552603	0.14371637
ARTN	3.36634748	3.68346415
NRTN	0.99943759	5.32786779
PSPN	0.5654626	1.3031218
LEFTY1	74.8549381	10.3490391
LEFTY2	21.6606487	6.43144534
AMH	2.24268026	3.72268063

Table S2: Cost of Activin A-based or small molecule-based protocols for DE generation

**One well of six-well plate (D1-D4)**							
Hogrebe et al, Nature Biotechnology, 2020							
name	Source	Final concentration	volume	Total amount	Unit price	price	Total price
Activin A	R&D systems; 338-AC-050	100ng/mL	8mL	800ng	$5.18/ug	$4.14	$4.17
CHIR99021	Cayman Chemical; 13122	3uM	2mL	6nmole	$5.23/umole	$0.03	
GiBi							
name	Source	Final concentration	volume	Total amount	Unit price	price	Total price
CHIR99021	Cayman Chemical; 13122	3uM	2mL	6nmole	$5.23/umole	$0.03	$0.04
Dorsomorphin	Selleck Chemicals; S7306	1uM	2mL	2nmole	$6.9/umole	$0.01	

Table S3: Pancreatic differentiation using different hPSC lines in this study and list of pancreatic progenitor efficiency at the end of stage 4 from the representative publications over the past five years.

<table>
<tr><th>Cell line</th><th>SOX17+% (D4)</th><th>FOXA2+% (D4)</th><th>PDX1+%</th><th>NKX6-1+%</th></tr>
<tr><td>H1 hESC</td><td>87.2</td><td>88</td><td>85.7 (D14)</td><td>70 (D14)</td></tr>
<tr><td>HUES8 hESC</td><td>80.8</td><td>92.5</td><td>99.5 (D10)</td><td>34.6 (D16)</td></tr>
<tr><td>IMR90C4 iPSC</td><td>68.4</td><td>83.8</td><td>64.5 (D10)</td><td>15 (D17)</td></tr>
<tr><td>H9 hESC</td><td>85</td><td>85</td><td>58 (D14)</td><td>-</td></tr>
<tr><td>6-9-9 iPSC</td><td>84.1</td><td>-</td><td>-</td><td>-</td></tr>
<tr><td>RUES2_GLR hESC</td><td>74.4</td><td>-</td><td>-</td><td>-</td></tr>
<tr><td>19-9-7 iPSC</td><td>75</td><td>-</td><td>-</td><td>-</td></tr>
<tr><th colspan="5">PDX1+NKX6.1+% (end of stage 4) in previous studies</th></tr>
<tr><td colspan="2">Nostro et al., 2015</td><td colspan="3">73.8%</td></tr>
<tr><td colspan="2">Millman et al., 2016</td><td colspan="3">52-88%</td></tr>
<tr><td colspan="2">Toyoda et al., 2017</td><td colspan="3">60.7%</td></tr>
<tr><td colspan="2">Memon et al., 2018</td><td colspan="3">68.4% (90.1% modified)</td></tr>
<tr><td colspan="2">Sharon et al., 2019</td><td colspan="3">70%</td></tr>
<tr><td colspan="2">Nair et al., 2019</td><td colspan="3">>70%</td></tr>
<tr><td colspan="2">Rosado-Olivieri et al., 2019</td><td colspan="3">55-61.2%</td></tr>
<tr><td colspan="2">Mahaddalkar et al., 2020</td><td colspan="3">40-50% (62.5% modified)</td></tr>
</table>

Table S4: Key reagents or resources used in this paper:

REAGENT or RESOURCE	SOURCE	IDENTIFIER
**Antibodies**		
Anti-SOX17, 1:200	R&D systems	Cat# AF1924<br>RRID: AB_355060
Anti-SOX17-APC, 1:50	R&D systems	Cat# IC1924A<br>RRID: AB_1964715
Anti-FOXA2, 1:500	Cell signaling Technology	Cat# 8186s<br>RRID: AB_10891055
Anti-PDX1, 1:500 (IF), 1:250 (FC)	R&D systems	Cat# AF2419<br>RRID: AB_355257
Anti-NKX6.1, 1:300 (IF), 1:400 (FC)	DSHB	Cat# F55A10-s<br>RRID: AB_532378
Anti-cTNT, 1:200	Thermal Scientific	Cat# MA5-12960<br>RRID: AB_11000742
Anti-MHC, 1:30	DSHB	Cat# MF20-s<br>RRID: AB_2147781
Anti-C-peptide, 1:300	DSHB	Cat# GN-ID4-s<br>RRID: AB_2255626
Anti-proglucagon, 1:1000	Cell signaling Technology	Cat# 8233<br>RRID: AB_10859908
Anti-NKX2.2, 1:300	DSHB	Cat#74.5A5<br>RRID: AB_531794
Anti-ISL1, 1:30	DSHB	Cat#39.4D5-s<br>RRID: AB_2314683
Anti-PAX6, 1:50	DSHB	Cat#PAX6<br>RRID: AB_528427
**Chemicals, Peptides, and Recombinant Proteins**		
iMatrix-511	Stemgent	Cat# NP892012
Y-27632 (hydrochloride)	Cayman Chemical Company	Cat# 10005583
CHIR99021	Cayman Chemical Company	Cat# 13122
Dorsomorphin	Sigma-Aldrich	Cat# P5499-5MG
Human Serum Albumin	Biological Industries	Cat# 05-720-1B
SANT-1	Tocris	Cat# 1974
Retinoic acid	Sigma-Aldrich	Cat# R2625-100MG
PDBu	Cell signaling Technology	Cat# 12808S
Nicotinamide	Sigma-Aldrich	Cat# N0636-100G
Recombinant human EGF	R&D systems	Cat# 236-EG-200
A83-01	Sigma-Aldrich	Cat# SML0788-5MG
Recombinant human FGF10	PeproTech	Cat# 10026
Recombinant human BMP4	R&D systems	Cat# 314-BP-010
Chemically Defined Lipid Concentrate	ThermoFisher	Cat# 11905031
Recombinant Activin A	R&D systems	Cat# 338-AC-050
Forskolin	Cayman Chemical Company	Cat# 11018
Wnt-C59	Selleck Chemicals	Cat# S7037
Fluo-4AM	Thermo Scientific	Cat#F14217
**Critical Commercial Assays**		
Human insulin Elisa kit	Alpco	Cat# 80-INSHU-E01.1
Direct-zol RNA MiniPrep Plus kit	Zymo research	Cat# R2071
Maxima First Strand cDNA synthesis kit	ThermoFisher	Cat# K1641
SYBR Green PCR master mix	ThermoFisher	Cat# 4367659
RNA clean & concentrator kit	Zymo Research	Cat# R1013
TruSeq Stranded mRNA library kit	illumina	Cat# 20020594
**Deposited Data**		
Raw and analyzed data	This study	GEO: GSE142572<br>GEO: GSE146985

Experimental Models: Cell Lines		
H9 (WA09) hESC line	WiCell Research Institute	NIH approval: NIHhESC-10-0062
H1 (WA01) hESC line	WiCell Research Institute	NIH approval: NIHhESC-10-0043
H1 OCT4-GFP hESC line	WiCell Research Institute	NIH approval: NIHhESC-10-0043
HUES8 hESC line	Harvard University	N/A
IMR90C4 human iPSC line	WiCell Research Institute	N/A
6-9-9 human iPSC line	WiCell Research Institute	N/A
19-9-11 human iPSC line	WiCell Research Institute	N/A
RUES-GLR reporter hESC line	Rockefeller University	N/A
SOX17-mCherry H9 hESC line	Dr. Ed Stanley lab, Murdoch Children Research Institute	N/A

Oligonucleotides		
**Gene name**	**Forward primer**	**Reverse primer**
GAPDH	GTGGACCTGACCTGCCGTCT	GGAGGAGTGGGTGTCGCTGT
NANOG	CGAAGAATAGCAATGGTGTGACG	TTCCAAAGCAGCCTCCAAGTC
SOX2	CAAGATGCACAACTCGGAGA	GTTCATGTGCGCGTAACTGT
OCT4	CAGTGCCCGAAACCCACAC	GGAGACCCAGCAGCCTCAAA
T	AAGAAGGAAATGCAGCCTCA	TACTGCAGGTGTGAGCAAGG
MIXL1	CGCCGGACCCGGTACCCCGA	CGCCGAGACTTGGCACGCCTGT
GSC	GAGGAGAAAGTGGAGGTCTGGTT	CTCTGATGAGGACCGCTTCTG
FOXA2	GGGAGCGGTGAAGATGGA	TCATGTTGCTCACGGAGGAGTA
SOX17	GGCGCAGCAGAATCCAGA	CCACGACTTGCCCAGCAT
AFP	CTTTGGGCTGCTCGCTATGA	GCATGTTGATTTAACAAGCTGCT
HNF4A	CGAAGGTCAAGCTATGAGGACA	ATCTGCGATGCTGGCAATCT
TBX3	CCCGGTTCCACATTGTAAGAG	GTATGCAGTCACAGCGATGAAT

Software and Algorithms		
ImageJ	Schneider et al., 2012	https://imagej.nih.gov/ij/
FlowJo V10		https://www.flowjo.com/solutions/flowjo/downloads
HISAT2	Kim et al., 2015	http://dx.doi.org/10.1038/nmeth.3317
Rpkmforgenes	Sandberg lab	http://sandberg.cmb.ki.se/media/data/rnaseq/instructions-rpkmforgenes.html
Gene-E		https://software.broadinstitute.org/GENE-E/
FastQC		http://www.bioinformatics.babraham.ac.uk/projects/fastqc/
Kallisto quant	Bray et al., 2016	http://dx.doi.org/10.1038/nbt.3519
Deseq2	Love et al., 2014	http://dx.doi.org/10.1186/s13059-014-0550-8
Limma-voom	Law et al., 2014<br>Liu et al., 2015	http://dx.doi.org/10.1186/gb-2014-15-2-r29<br>http://dx.doi.org/10.1093/nar/gkv412
Morpheus		https://software.broadinstitute.org/morpheus
Metascape	Zhou et al., 2019	http://metascape.org/gp/index.html#/main/step1
Prism8 Graphpad		https://www.graphpad.com/scientific-software/prism/

Video S1. Sox17-mCherry H9 cells were treated with 5 μM CH in RPMI for 24 hours and then cultured in RPMI plus B-27 minus insulin supplement for two days. On day 3, cells were treated with 2 μM Wnt-C59 for two days, followed by media change to RPMI plus B-27 supplement from day 5. Video of beating cardiomyocytes was taken on day 9.

Video S2. RUES2_GLR cells (SOX17-tdTomato) were treated with 3 μM CH in RPMI for 24 hours and then cultured in RPMI with B27 minus insulin supplement. Time-lapse imaging was performed from day 2 to day 3 of differentiation.
